# Post-COVID Syndrome in Adults—An Overview

**DOI:** 10.3390/v15030675

**Published:** 2023-03-04

**Authors:** Rüdiger E. Scharf, Juan-Manuel Anaya

**Affiliations:** 1 Current Address: Department of Medicine, Division of Cardiology, Angiology, Hemostasis and Internal Intensive Care Medicine, University Medical Center Mannheim, University of Heidelberg, D-68167 Mannheim, Germany; 2 Harvard Medical School, Program in Cellular and Molecular Medicine, Boston Children’s Hospital, Karp Family Research Laboratories, Boston, MA 02115, USA; 3 Institute of Transplantation Diagnostics and Cell Therapy, Division of Hemostasis, Hemotherapy and Transfusion Medicine, Heinrich Heine University Medical Center, D-40225 Düsseldorf, Germany; 4 Current Affiliation & Address: National Academy of Medicine of Colombia, Bogotá 110221, Colombia; 5 Health Research and Innovation Center at Coosalud, Cartagena 130001, Colombia

**Keywords:** post-COVID syndrome, hyperinflammation, thrombo-inflammation, hypercoagulability, immunocompromised condition, autoimmunity, psychosocial implications, vaccination

## Abstract

This article provides an overview of various aspects related to post-COVID syndrome. Apart from its prevalence, symptoms and sequelae, risk determinants, and psychosocial implications, the pathogenesis of post-COVID condition is discussed in more detail. A focus on thrombo-inflammation in SARS-CoV-2 infection, the role of neutrophil extracellular traps, and the prevalence of venous thromboembolism is made. Moreover, COVID-19 and post-COVID syndrome in immunocompromising conditions, and the impact of vaccination on the prevention and treatment of post-COVID symptoms are reviewed. Autoimmunity is a hallmark of post-COVID syndrome, and, therefore, is another focus of this article. Thus, misdirected cellular and humoral immune responses can enhance the risk of latent autoimmunity in post-COVID syndrome. Facing the high prevalence of COVID-19 cases worldwide, it can be assumed that autoimmune disorders will increase globally over the next few years. Recent advances in identifying genetically determined variants may open the avenue for a better understanding of the susceptibility to and severity of SARS-CoV-2 infection and post-COVID syndrome.

## 1. Introduction

Coronavirus disease 2019 (COVID-19), caused by the severe acute respiratory syndrome coronavirus-2 (SARS-CoV-2), is a clearly defined disorder. By contrast, the post-COVID syndrome is currently variable in its definition, representing a heterogeneous condition that develops in patients with prior SARS-CoV-2 infection. The spectrum of symptoms and sequelae after confirmed (or probable) COVID-19 is wide and variable. Currently, more than 100 symptoms have been reported in individuals with post-COVID condition, all of which have in common that they can have an unfavorable impact on quality of life, physical and/or mental activity, vitality, or resilience of affected subjects. Remarkably, the post-COVID syndrome in children is different from that of adults in its prevalence, manifestation, and characteristics [1,2].

## 2. Defining Post-COVID Syndrome

The post-COVID syndrome includes a large range of different health complaints, symptoms, or sequelae that arise following acute SARS-CoV-2 infection. Due to heterogeneity of symptoms and inconsistent assignment, no uniform terminology exists, and a number of clinical definitions for the post-COVID condition have been proposed (Table 1). Some definitions restrict the post-COVID-9 syndrome exclusively to symptoms beyond 12 weeks, while others define it by inclusion of symptoms related to the development of new, returning, or ongoing complaints as “post-acute” sequelae of the SARS-CoV-2 infection beyond four weeks [3].

Currently, no universally accepted definition of the post-COVID syndrome exists. However, implementation of a globally consented, uniform, and approved terminology and definition of the post-COVID syndrome is critical to its proper diagnosis and corresponding treatment in clinical practice, but also relevant for clinical research (e.g., study design, data analysis, assessment of treatment) and correct scientific evaluation.

## 3. Prevalence of Post-COVID Syndrome

The combined prevalence of new or persistent symptoms at four or more weeks from acute SARS-CoV-2 infection (CDC definition) is estimated to be about 43% [3,10]. The corresponding rate among hospitalized patients, including those admitted to the intensive care unit, is approximately 54%, and around 34% among non-hospitalized subjects [10]. At 12 weeks or more, up to 20% of outpatients after confirmed SARS-CoV-2 infection suffer from post-COVID syndrome. These data are derived from meta-analyses across 50 studies that had been performed early in the pandemic, enrolling more than 880,000 or 1,700,000 persons [3,10].

However, due to heterogeneity in definition, uncertainty in proper diagnostic assignment, and differences in study design (e.g., recruitment strategies, duration of follow-up, and study sample size), the reported rates should be considered with caution. Two other systematic reviews and meta-analyses enrolling more than 250,000 patients of the COVID-19 population, did not overcome the inaccuracies and imprecision caused by heterogeneity in definition and study design in 63 or 45 eligible publications, respectively [11,12].

More contemporary data among adults who have had triple vaccination suggest that between 4.2% and 5.0% of individuals self-reported having symptoms of the post-COVID syndrome beyond 12 weeks after confirmed SARS-CoV-2 infection [13]. In addition, current evidence indicates that, using a 12-week criterion for symptoms, approximately 2% to 10% of vaccinated persons infected with present SARS-CoV-2 variants suffer from post-COVID symptoms [3]. Therefore, in times of widespread vaccination and emerging variants causing less critical illness than earlier SARS-CoV-2 species, it is concluded that the post-COVID syndrome may now be less frequent (and less pronounced) after acute infection.

## 4. Symptoms and Sequelae of Post-COVID Syndrome

More than 100 symptoms have been reported among persons with post-COVID condition. The most frequently observed symptoms are fatigue (23%, 95% CI 17–30), memory deficits (14%, 95% CI 10–19), dyspnea (13%, 95% CI 11–15), depression (13%, 95% CI 3–15), anxiety (11%, 95% CI 5–25), anosmia (12%, 95% CI 6–23), sleep problems (11%, 95% CI 5–23), and joint pains (10%, 95% CI 4–22) [10]. Less frequent is tachycardia (6%, 95% CI 3–11) [10].

These data are derived from a large systematic review and meta-analysis across 50 studies to estimate the individual complaints and symptoms with post-COVID syndrome [10]. The meta-analysis included 4165 non-hospitalized subjects (from five studies), 67,161 hospitalized patients (from 22 studies), and 1,608,677 SARS-CoV-2 positively tested persons (from 23 studies without reported hospitalization status). Of note, most of the above indicated prevalences of symptoms result from combined analysis upon pooling of hospitalized and non-hospitalized persons from corresponding studies.

**Figure 1:**
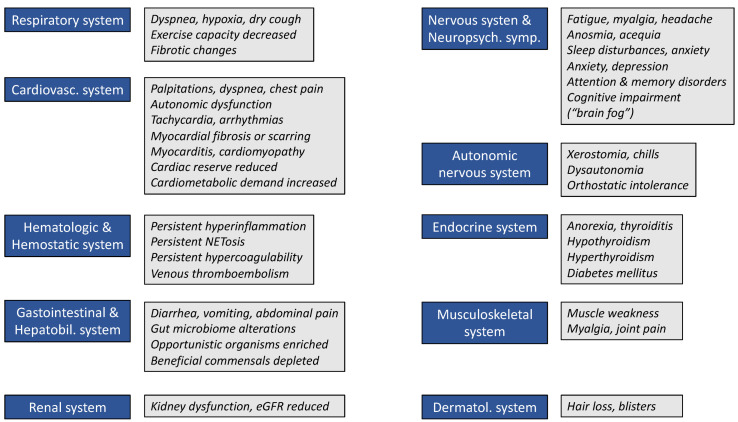
Common symptoms and sequelae of post-COVID syndrome that are assigned by organ system. Abbreviation: eGFR, estimated glomerular filtration rate (decreased when <90 mL/min).

Some of the symptoms displayed in Figure 1 are likely to be due to dysautonomia [14]. Autonomic dysfunction appears to be a rather frequent feature of the post-COVID condition and can cause, for example, orthostatic intolerance syndromes, neurogenic orthostatic hypotension, labile blood pressure, postural orthostatic tachycardia syndrome [15], and other more non-specific sequelae, such as gastrointestinal symptoms (e.g., abdominal pain, vomiting, constipation, or loose stools), flushing, sweating abnormalities, or temperature intolerance [14].

Moreover, emerging evidence suggests that a still unknown proportion of individuals will develop chronic disease conditions, such as diabetes [16], autoimmune diseases [17], cerebrovascular and/or cardiovascular disorders [18], including venous thromboembolism [19], psychological distress [20], or mental illness [21], all of which subsequently contribute to the burden of persisting symptoms and sequelae following acute SARS-CoV-2 infection. Post-COVID condition may affect the respiratory system (e.g., lung fibrosis), the cardiovascular system (e.g., myocarditis, heart failure, infarction, and arrhythmias), the musculoskeletal system (e.g., post-viral arthritis), and the nervous system (e.g., stroke, chronic fatigue syndrome, anosmia, and ageusia).

## 5. Pathogenesis of Post-COVID Syndrome

**Figure 2:**
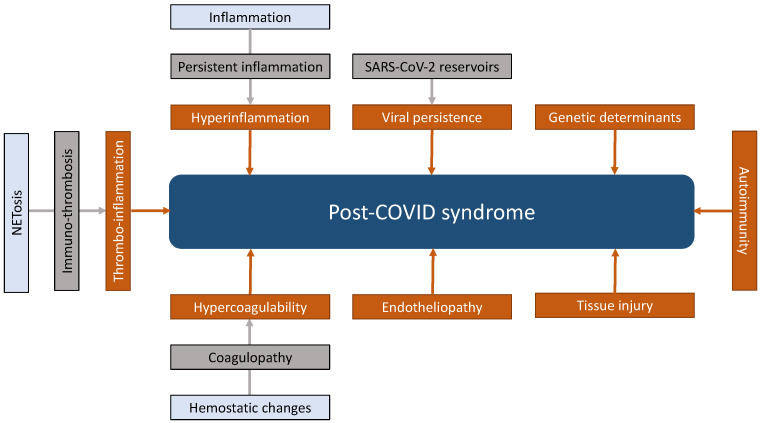
Proposed mechanisms related to the development and manifestation of post-COVID syndrome. Depicted are SARS-CoV-2-dependent and viral-independent pathologies in eight different domains. Of note, inflammation and thrombosis are tightly connected processes which are termed “immuno-thrombosis”. Dysregulated and excessive activation of immune-thrombosis leads to “thrombo-inflammation”, causing tissue ischemia by microvascular and macrovascular thrombus formation. Abbreviation: NETosis, formation of intravascular neutrophil extracellular traps.

Several distinct mechanisms are involved in the pathogenesis of acute COVID-19. Basically, some of them, if not all, are believed to cause post-COVID syndrome or, at least, contribute to the manifestation of post-COVID condition in its various clinical phenotypes. However, despite this contention, the exact pathogenesis and interactions among various pathomechanisms involved are, to a large extent, still incompletely understood. Overall, recent evidence suggests that both viral and host factors in combination lead to post-COVID syndrome, thereby interacting with each other and culminating into a common pathology. Such a conceptual approach allows linking SARS-CoV-2 infection to direct cellular damage, tissue injury, and structural organ damage and dysfunction with the development of post-COVID syndrome. In accord with this concept, viral-dependent and viral-independent mechanisms can be categorized to define eight different domains that are synoptically depicted in Figure 2.

### 5.1. Predominant Pathomechanisms of Acute COVID-19

The cell surface angiotensin-converting enzyme 2 (ACE2) receptor is the target for SARS-CoV-2 binding, cellular invasion, and infection [22] This receptor is widely spread in multiple organs, explaining the multisystemic nature of the disease. Upon the CD4 and CD8 response and regulated inflammatory reaction of the immune system, the infection is controlled, and most of individuals experience uncomplicated recovery. By contrast, various mechanisms, including (1) maladaptation of the ACE2 pathway, (2) dysregulation of the immune system leading to (3) hyperinflammatory responses with highly increased levels of interleukin-1β (IL-1β), IL-2, IL-6, IL-10, and tumor necrosis factor-α (“cytokine storm”), (4) direct viral toxicity, (5) endothelial damage and microvascular injury, and (6) hypercoagulability with resulting (7) micro- and macrothrombosis, are responsible for severe life-threatening COVID-19 [23,24].

### 5.2. Pathomechanisms Contributing to Post-COVID Syndrome

Proposed underlying mechanisms related to the development and manifestation of post-COVID syndrome can include (1) viral persistence upon initial infection of host cells by SARS-CoV-2, (2) cellular damage due persistent hyperinflammation, (3) immunologic aberrations caused by molecular mimicry, epitope spreading, bystander activation, or superantigen, and (4) hemostatic changes, predominantly characterized by coagulation abnormalities [23,24,25,26,27,28]. Some of these mechanisms and abnormal or misdirected drivers are displayed in Figure 1 and discussed in more detail below.

#### 5.2.1. Viral Persistence and Abnormalities in Adaptive Immunity Including Autoimmunity

Several investigators have observed pulmonary and gastrointestinal SARS-CoV-2 reservoirs that can persist for up to four months [25,29]. Moreover, in a recent autopsy study on 44 patients, persistent SARS-CoV-2 RNA was detected outside the respiratory tract in multiple anatomic sites, including the brain, as late as 230 days following onset of COVID-19 symptoms [30]. A hypothesis-driven model of viral persistence has been proposed for the evolution and manifestation of post-COVID syndrome [31]. This model is based on increased proinflammatory cytokine production that results from the persistence of the SARS-CoV-2 virus or one of its molecular components (e.g., the spike protein).

Viral persistence also triggers long-term stimulation of the adaptive immune system, thereby activating autoreactive T lymphocytes through antigen-presenting cells. For example, T cell-mediated abnormalities in post-COVID syndrome can become evident by autoimmune thyroid dysfunction [27]. Likewise, persistent B cell activation and subsequent production of autoantibodies that are detected in more than 50% of patients with post-COVID condition can induce a variety of autoimmune disorders (e.g., antiphospholipid syndrome, lupus erythematosus, thyroid disease, among others, see Section 5.2.4.1.) [17,25,26,27,28].

Conversely, in more than 40% (range, 42–53%) of patients with post-COVID syndrome, no anti-SARS-CoV-2 antibodies are detectable; however, these individuals are capable of exhibiting SARS-CoV-2-specific T cell responses [32].

#### 5.2.2. NETosis: A Structural and Functional Link in COVID-19 between Innate Immunity, Inflammation, Endothelial Dysfunction, Hemostasis, and Thrombosis

Cellular players of the innate immunity are in the frontline to attack infections. Upon invasion, microorganisms can activate neutrophils, monocytes, tissue macrophages, and endothelial cells, thereby triggering the production and release of proinflammatory cytokines and stimulating serine proteases of the coagulation cascade. Inflammation and activation of hemostasis are concomitant responses of the host defense to contain invaded pathogens. Thereby, complex processes and interactions between cellular and humoral components occur, nowadays designated “immuno-thrombosis” and referred to as “thrombo-inflammation” in case of excessively activated or dysregulated immune-thrombosis (Figure 2) [33]. For example, pathogen-induced loss of normal antithrombotic and anti-inflammatory endothelial cell functions causes dysregulation of coagulation, platelet activation, recruitment of leukocytes, and unleashing of the complement system in the microvasculature and results in microthrombosis with subsequent hypoxia [34].

Upon activation by invaded microorganisms, neutrophils expel their condensed nuclear chromatin and form intravascular neutrophil extracellular traps (NETs). NETs are meshwork-like structures composed of DNA, histones, and neutrophil-derived granule proteases that can kill bacteria [35,36]. The formation of NETs (NETosis) is an evolutionary mechanism to capture invaded pathogens and limit their systemic spreading by containment [37]. NET formation and immune-thrombosis are also involved in host defense of viral diseases, specifically in SARS-CoV-2 infection [33,34,38,39], but can also occur under sterile conditions [40].

Over the past decade, the pathological role of NETosis has become evident since this mechanism is crucially involved in a variety of clinical conditions, including acute or chronic inflammatory and autoimmune disorders, atherosclerosis, thrombo-occlusive diseases in the venous and arterial circulation, ischemia/reperfusion injury, age-related tissue fibrosis, diabetes, cancer, and malignant hematological disorders [33,34].

In line with the above consideration, these clinical settings and pathologies have in common that they can now be defined as thrombo-inflammatory processes. Correspondingly, aberrant crosstalk between inflammation and thrombosis within formed NETs can have severe consequences. This is particularly true with respect to COVID-19 and the impact of SARS-CoV2 and neutrophils on endothelium and hemostasis. The secretion of proteolytic enzymes the release of reactive oxygen species can damage endothelial cells, perturb their thromboresistance, and thereby promote thrombogenesis [34]. Infection of endothelial cells upon entry of SARS-CoV-2 via ACE receptor causes an endotheliopathy (“endotheliitis”), which is associated with a prominent increase in von Willebrand factor (VWF) and factor VIII:C (FVIII:C) levels, as reported in several small case series [41,42,43]. Again, these changes promote arterial and venous thrombosis.

NETs can also interact with adhesive proteins (e.g., VWF) and form a scaffold for the activation of platelets and the coagulation system, thereby bolstering their prothrombotic properties, promoting thrombin generation, and fostering nascent thrombi [33,34,35].

#### 5.2.3. Hemostatic Abnormalities in COVID-19 and Post-COVID Syndrome

Profound hemostatic alterations can occur during or following an acute SARS-CoV-2 infection, and also be present in post-COVID syndrome. The abnormalities preferentially affect the coagulation system rather than the megakaryocyte-platelet system and are associated with a high risk of thromboembolic complications that manifest as micro- and macrothrombi.

##### 5.2.3.1. Prevalence of Thrombotic Complications

The cumulative incidences of venous thromboembolism (VTE) in hospitalized patients with COVID-19 during a 7-day and 30-day follow-up were 16% and 42%, respectively, and significantly higher among patients in the intensive care unit (ICU) (day 7, 26%; day 30, 59%), as reported in a representative Dutch study from the early phase of the pandemic [44]. Similar findings, also from the early phase of the pandemic, were obtained in a meta-analysis in which the prevalence of VTE was expressed as weighted mean prevalence [45]. A subsequent meta-analysis documented an estimated overall VTE prevalence of 14.1% (95% CI 11.6–16.9) and a prevalence of 45.6% (95% CI 31.0–66.2) for patients in the ICU [46]. Similar prevalences were reported by other investigators [47,48,49] (Table 2).

Another meta-analysis, evaluating the rate of VTE among COVID-19 patients within the first 90 days of hospital discharge, showed that cumulative incidences range between 0.2% and 14.8% [50]. Using a random effect model, the pooled incidence of post-COVID VTE is 1.8 (95% CI, 0.8–4.1), which appears to be higher than in other groups of seriously ill patients.

In addition to VTE, the prevalence of arterial thrombotic events, such as myocardial and cerebral infarction, is also increased among patients with COVID-19 (up to 3%, 95% CI 2–5) [46,48]. Importantly, due to persistently low levels of protein C, subsequently reduced generation of activated protein C, and resulting hypercoagulability (see Section 5.2.3.2.), the risk of ischemic stroke may remain increased after hospital discharge and in patients with post-COVID syndrome [51]. Moreover, individuals with inherited thrombophilia (e.g., factor V Leiden, prothrombin G20210A mutation) have a higher risk of VTE following SARS-CoV-2 infection than those without genetic variations [49]. However, across countries, there is a wide variation in the incidence of venous and arterial thrombotic events after a COVID-19 diagnosis [52]. This variation highlights the challenges in combining estimates and analyzing data from different health care systems and with different public health policies [53].

##### 5.2.3.2. COVID-19-Associated Coagulopathy—A Hypercoagulable Condition

The coagulation abnormalities observed in COVID-19 are consistent with a systemic disorder, reflecting infection-induced inflammatory changes (‘acute phase reaction’) and hyperinflammatory responses [54,55]. By contrast to other pathogens, SARS-CoV-2 does not appear to have intrinsic procoagulant effects itself [55].

Typical coagulation findings in COVID-19 are greatly elevated levels of fibrinogen, D-dimer, and fibrin/fibrinogen degradation products, all of which can correlate with the disease severity and prognosis [54]. Some of the laboratory features can resemble those observed in sepsis-induced disseminated coagulation (DIC); however, patients with COVID-19 have no bleeding manifestations and thus do not fulfill the criteria of DIC [54,55]. To make a clear distinction between both phenotypes, the term COVID-19-associated coagulopathy (CAC) has been introduced. However, the nature of CAC is still incompletely understood [51].

COVID-19 can also cause a dysregulation of the protein C (PC) system, resulting in low levels of activated PC (APC) [51]. APC is an intrinsic anticoagulant. Deficiency or dysfunction in this protective system contributes to hypercoagulability in COVID-19 and post-COVID syndrome.

Other relevant abnormalities related to the prothrombotic state of SARS-CoV-2 infection include greatly elevated VWF and FVIII:C levels (see Section 5.2.2). Importantly, regulation of the VWF structure can also be affected in COVID-19, resulting in an abnormally high proportion of large-sized VWF multimers which display prothrombotic function [33].

Moreover, multiple case reports have documented that patients with COVID-19 can develop antiphospholipid antibodies [51]. Similar to “classic” antiphospholipid syndrome, these autoantibodies are directed against several serine proteases of the coagulation system and cause their activation. This observation is in accord with the clinical phenotype, which involves thrombosis in arteries, veins, and the microcirculation. Generation of antiphospholipid antibodies is another evidence of autoimmunity in COVID-19. Importantly, antiphospholipid antibodies can persist and represent a distinct feature of post-COVID syndrome. Therefore, it is highly recommended to monitor antiphospholipid antibody levels in this group of patients.

##### 5.2.3.3. Platelet Dysfunction in COVID-19

Inconsistent findings have been reported on platelet activity ex vivo and their responsiveness in vitro among COVID-19 patients with or without thromboembolic events. The conflicting results are due, at least in part, to heterogeneity in patient populations, disease state, study design, and differences in methods among the studies. Several investigators demonstrated a prothrombotic platelet phenotype in SARS-CoV-2 infection [33,51]. As discussed above, such an observation would correspond to cellular interactions in thrombo-inflammation, in which a mutual activation of platelets and neutrophils is a crucial event.

However, in recent studies, platelets from COVID-19 patients were shown to exhibit impaired aggregation and impaired activation of the responsible integrin receptor αIIbβ3 (GPIIb-IIIa) upon agonist-induced stimulation in vitro [56,57]. The reduced responsiveness of platelet functions ex vivo was not due to prior platelet activation in vivo. Interestingly, in one of the studies, a significantly increased proportion of circulating platelet-neutrophil conjugates was reported [56]. This observation reflects the interplay between platelets and neutrophils which is mediated by binding of platelet GPIbα to the MAC1 receptor (integrin αMβ2) on neutrophils.

#### 5.2.4. Post-COVID Syndrome and Autoimmunity

Patients with COVID-19 have a significantly increased risk of developing autoimmune diseases after SARS-CoV-2 infection. This conclusion has been drawn by numerous investigators worldwide, reporting that patients with acute illness produce multiple autoantibodies, which can lead to various autoimmune disorders and be associated with disease severity and mortality [58,59,60]. However, until recently, no large-scale population-based analysis was available to confirm the association with autoimmune diseases.

##### 5.2.4.1. Epidemiology

A large retrospective cohort study published in February 2023 now provides evidence about this association and documents a different degree of risk for distinct autoimmune diseases following acute SARS-CoV-2 infection [61]. For their study, Chang et al. used the TriNetX database, a multi-institutional US Collaborative Network, which incorporates 48 international healthcare organizations and holds by far the largest set of COVID-19 data worldwide.

To reduce detection bias, the investigators only included adult patients who had at least two healthcare visits and received PCR testing (index date) during the study period. Individuals who had been vaccinated against SARS-CoV-2 and those who were diagnosed with autoimmune disease or neoplasm before or within 30 days after index date were excluded to reduce protopathic bias. Of the initially enrolled study participants (n = 5,913,210), more than 888,000 patients were assigned to the COVID-19 cohort and more than 2,900,000 individuals to the non-COVID-19 cohort (composed of participants who were tested negative by PCR and were not diagnosed with COVID-19 throughout the follow-up period). Finally, 887,455 COVID-19 patients and 887,455 non-COVID-19 control subjects were propensity score-matched (1:1) for age, sex, race, adverse socio-economic status, lifestyle-related variables, and comorbidities. (A statistical analysis based on propensity scores matching can strengthen the assessment of a causal relationship and minimize the bias due to confounding variables). Both cohorts were retrospectively analyzed for the incidence of newly recorded autoimmune diseases as primary endpoint [61].

The investigators now demonstrate that, at a 6-month follow-up period, the risk of various autoimmune diseases is significantly higher in the COVID-19 cohort than in the non-COVID population after accounting for competing risk of death [61]. The autoimmune disorders include rheumatoid arthritis, ankylosing spondylitis, systemic lupus erythematosus, dermatopolymyositis, systemic sclerosis, Sjögren’s syndrome, mixed connective tissue disease, Behçet’s disease, polymyalgia rheumatica, vasculitis, psoriasis, inflammatory bowel disease, celiac disease, and type-1 diabetes mellitus. Of note, the risk of mortality is also increased. Statistical details of the study are displayed in Table 3.

##### 5.2.4.2. COVID-19—A Trigger or Cause for Autoimmune Diseases

The large propensity scores matched the study by Chang and colleagues convincingly confirms the association of COVID-19 with autoimmune diseases [61]. However, the definite mechanisms by which autoimmunity and subsequent pathologies are triggered or caused during or after SARS-CoV-2 infection remain unknown. Generally, viruses can trigger autoimmunity through different mechanisms, including molecular mimicry [62], epitope spreading [63], bystander activation [64], or superantigen [65].

Currently, it is hypothesized that prolonged inflammation and/or hyperinflammatory host responses to SARS-CoV-2 can trigger the adaptive immune system to produce antibodies against viral antigens that share structural similarities with self-antigens [61]. The dysregulation and malfunction of the immune system can result in a loss of self-tolerance to self-antigens [66]. These basic considerations are in accord with numerous clinical studies on patients with acute and post-acute conditions in COVID-19 and systematic reviews [17,58,67,68,69].

##### 5.2.4.3. Autoimmunity—A Hallmark of Post-COVID Syndrome

Hospitalized patients with acute SARS-CoV-2 infection can produce multiple autoantibodies that may have an impact on the severity and outcome of COVID-19, as reported in a monocenter study [58]. Persistent immune activation, as characterized by increased levels of helper T cell 9 (Th9), CD8^+^ effector T cells, naive B cells, and CD4^+^ effector memory T cells, and persistent proinflammatory responses, as evident from long-term elevation of various cytokines, contribute to the development of latent autoimmunity, latent poly-autoimmunity, or even overt autoimmunity, all of which can be a characteristic feature of patients with post-COVID syndrome [17,68].

Facing the global scale of the pandemic, and given the current evidence on latent autoimmunity in post-COVID condition, a significant increase in the incidence of autoimmune disorders it can be anticipated worldwide in the coming years [17,28].

It must be emphasized that only a proportion of patients with SARS-CoV-2 infection will develop post-COVID syndrome with or without autoimmune diseases. This proportion can vary tremendously depending on the respective study population [67]. Moreover, as discussed below (see Section 8.), vaccination may have a beneficial impact on post-COVID syndrome and treatment, attenuation, or prevention of autoimmunity.

## 6. COVID-19 and Post-COVID Syndrome in Immunocompromised Hosts

Immunocompromised individuals due to cancer, hematologic malignancies, solid-organ transplantation, innate immunodeficiency, or other causes of immunosuppression (e.g., people living with HIV or those receiving immunomodulatory agents for treatment of autoimmune disease) are at presumed risk of more severe COVID-19, hospitalization, and death from complications of SARS-CoV-2 infection. Indeed, a more recent review has documented that subjects with immunocompromising conditions are at increased risk of critical course, longer hospitalization, and lethal outcome of COVID-19 [70]. This holds specifically true for certain SARS-CoV-2 variants, including B.1.1.7 (alpha), B.1.351 (beta), P1 (gamma), and B1.6172 (delta) [71]. Moreover, immunocompromising conditions are associated with decreased protective immunity after an initial infection with SARS-CoV-2 and exhibit decreased efficacy of COVID-19 vaccines. Remarkably, SARS-CoV-2 infection itself can induce immunodeficiency in recovered patients by downregulating CD19 expression in B cells [72].

Conversely to the above indicated increased risk, immunocompromised individuals may also exhibit less detrimental inflammatory responses than previously healthy subjects upon SARS-CoV-2 infection. Remarkably, immunocompetent children appear to be significantly more affected by post-COVID syndrome than those with immunocompromised conditions [73]. A potential explanation for this finding of less frequent and milder post-COVID syndrome among immunocompromised children may be their less serious course of COVID-19. However, the observation requires further examination to disentangle the underlying mechanism(s).

## 7. Risk Determinants Associated with Post-COVID Syndrome

Based on studies analyzing data from the early phase of the pandemic, it had been suggested that several biological markers, symptoms, clinical findings, or baseline risk factors, such as comorbidities, may identify or predict those patients who will develop post-COVID syndrome [74]. However, the available evidence had generally been of poor quality. Since then, specifically, the predictive value of biomarkers is subject of debates. To overcome some of the controversies, multi-dimensional immune phenotyping in conjunction with unbiased machine learning may be a promising approach, as demonstrated recently in a small exploratory, cross-sectional study [75]. Importantly, marked differences in myeloid cells, lymphocyte populations, and humoral responses were noted among patients relative to matched control groups.

Recently, a large retrospective matched cohort study using a UK-based database investigated symptoms that are associated with confirmed SARS-CoV-2 infection beyond 12 weeks in non-hospitalized adults and risk factors that are being associated with developing persistent symptoms [76]. For the analysis of outcomes, including 115 symptoms and post-COVID syndrome (defined as a composite outcome of 33 symptoms by the WHO clinical case definition), more than 486,000 adults with confirmed SARS-CoV-2 infection and about 1,945,000 propensity score matched adults with no recorded evidence of SARS-CoV-2 infection were selected. Among the cohort of patients infected with SARS-CoV-2, risk factors that were significantly associated with the post-COVID syndrome (*p* < 0.05) included female sex, belonging to an ethnic minority, socio-economic deprivation, smoking, obesity, and a wide range of comorbidities [76]. The risk of developing post-COVID syndrome was also found to be increased along with a gradient of decreasing age. Subsequently, similar results were obtained in a meta-analysis by investigators from the Ontario COVID-19 Science Advisory Table, who identified nine potential risk factors in association with post-COVID syndrome; additional indicators were invasive ventilation and days of hospitalization [3]. Thus, emerging evidence suggests that (1) female gender, (2) socio-economic conditions, (3) health determinants, and (4) comorbidities appear to be relevant independent risk factors for the post-COVID syndrome.

## 8. Impact of Vaccination on the Prevention and Treatment of Post-COVID Syndrome

Vaccination against SARS-CoV-2 is the leading strategy to combat the COVID-19 pandemic effectively and sustainably. Thus, various COVID-19 vaccines were shown to have a protective potential against SARS-CoV-2 in real-world settings, and to decrease the risk of severe illness [77]. However, increasingly, vaccinated individuals are being diagnosed with COVID-19 as a result of breakthrough SARS-CoV-2 infection [78,79]. Recently, Notarte et al. performed a systematic review, analyzing 17 studies selected from more than 2500 reports [80]. The authors assessed the impact COVID-19 vaccination both on the risk of developing of post-COVID syndrome and on already existing post-COVID symptoms among populations previously infected by SARS-CoV-2.

### 8.1. Breakthrough SARS-CoV-2 Infection in Vaccinated People

Six studies with a vaccine-infection-post-COVID-syndrome design, enrolling more than 17,250,000 individuals, documented that vaccination is associated with a reduced risk to develop post-COVID symptoms [80]. There was a high heterogeneity in the time from vaccination to infection. Two doses of vaccines were more effective than a single shot [81]. Interestingly, BNT162b2 (Pfizer/BioNTech) and mRNA-1273 (Moderna) vaccines were more effective for reducing the risk of post-COVID symptoms compared to Ad26.COV2.S (Janssen) [82].

### 8.2. Impact of Vaccination on Post-COVID Symptoms

Eleven studies investigated changes in post-COVID-19 symptoms among more than 36,700,000 persons after vaccination (infection-post-COVID-syndrome-vaccine design). Seven studies documented an improvement, while four articles reported on no change or even worsening in post-COVID symptoms following vaccination [80].

Among these 11 studies, 3 of them deserve further discussion. Ayoubkhani et al. found a 12.8% reduction in the odds of reporting post-COVID symptoms immediately after the first vaccine dose, but this reduction was not sustained over the following 12 weeks [83]. After a second dose, an 8.8% reduction in the odds of post-COVID syndrome was sustained over the next 9 weeks. The authors assumed inadequate immune responses as a reason for the lacking sustained effect after the first dose [83]. Tran and colleagues reported that vaccination can double the remission rate of post-COVID syndrome [84]. At 120 days, 16.6% of vaccinated participants indicated complete resolution of symptoms in comparison with 7.5% of those who were unvaccinated. The difference was significant [84]. Tsuchida et al. measured antibody titers and evaluated symptoms after vaccination following SARS-CoV-2 infection in a small study on 42 adults: 61% reported no change in symptoms, 21% worse symptoms, and 16% improvement of symptoms [85]. Interestingly, the investigators found significantly higher antibody titers in the group reporting worse symptoms compared to those with no change or improvement in symptoms. The authors hypothesize that an excessive immune response induced by the vaccine may be responsible [85].

Overall, the mechanisms by which vaccination against SARS-CoV-2 might attenuate symptoms in subjects with post-COVID syndrome are unclear. Given the uncertainty around the true effect of vaccines relative to natural recovery from post-COVID symptoms, a convincing explanation for how vaccines might reduce the multisystem manifestation of post-COVID syndrome is currently lacking.

## 9. Genetic Basis of the Susceptibility to and Severity of COVID-19

Human genetic variants can have an impact on the susceptibility to and severity of infectious diseases, as demonstrated for a number of human-tropic viruses [86,87]. This experience is also confirmed in SARS-CoV-2 infection [88,89]. However, the exact role of human genetics in determining clinical responses to SARS-CoV-2 has remained unclear until recently. Specifically, it is unknown why some individuals stay uninfected despite viral exposure, whereas other healthy young adults experience life-threatening COVID-19. Thus, SARS-CoV-2 infections display tremendous inter-individual variability, ranging from asymptomatic to lethal course of disease.

To address and possibly solve the enigma, the COVID-19 Host Genetic Initiative recently performed an enormously large study to identify genetic variants that account for the variability in individuals’ susceptibility to SARS-CoV-2 and in the severity of COVID-19 [90,91]. In this comprehensive analysis, the genomes of more than 49,500 individuals with COVID-19 (including approximately 6200 critically ill patients) were compared with the genomes of around 2 million control individuals without known infection. The comparison pointed to 13 loci: variants in 4 of these loci are associated with susceptibility to COVID-19, whereas variants in 9 others are associated with disease severity [90].

No such associations are specifically known for post-COVID condition. Conversely, global efforts are currently made to disentangle and dissect the human genetic basis of resistance to SARS-CoV-2 infection [92].

## 10. Psychosocial Impact of COVID-19 and Post-COVID Syndrome

A different aspect of the post-COVID condition is provided by sociological, socio-economic, and psychological studies, analyzing pandemic-related effects on society. Specific objectives of those explorations include the SARS-CoV-2-induced coercive deceleration effects of public and personal life, and their impact on behavior in health and disease. In this context, Hartmut Rosa, a German sociologist and philosopher who systematically analyzed continuous acceleration and alienation in modern societies, has referred to the coronavirus as “Inbegriff einer monströsen Unverfügbarkeit” (“epitome of a monstrous unavailability”) [93].

Indeed, the pandemic has had immediate effects on almost all strata of the society, ethnic groups, their social life, and life style (induced by social distancing, shut down, deceleration of public and/or personal activities, and nationwide lockdowns) [93]. Among the scientific community, multiple quite opposite changes as a consequence of the pandemic and resulting alterations in working conditions occurred [94]. For example, while the overall (writing) productivity increased, most scientists experienced a decrease in (laboratory) research activity.

Along with enormously high infectivity and fatality rates, COVID-19 has provoked universal psychosocial long-term effects by causing mass fear (“coronaphobia”), economic burden, and financial losses [95]. It is also evident that COVID-19 itself multiplied by forced quarantine to combat SARS-CoV-2 can induce obsessive behavior (e.g., anxiety, acute panic and hysteria, paranoia, and depression) [95].

In a similar way, post-COVID syndrome can disrupt the usual lifestyle of affected persons, and cause or exacerbate mental distress or illness [20]. Moreover, the post-COVID condition may contribute to increase present effects of the pandemic on social inequalities with respect to gender, ethnicity, education, labor market, and professional activities [96].

## 11. Conclusions, Future Directions, and Perspectives

The multi-organ manifestations and sequelae of COVID-19 beyond the acute phase of SARS-CoV-2 infection are recognized as a novel disease entity. However, both the pathophysiology and prevalence of post-COVID syndrome are still puzzling and incompletely understood. This, in part, is due to the highly variable clinical spectrum of symptoms which abate or, rather uncommonly, aggravate or exacerbate over time. Consequently, prospective longitudinal clinical studies are required to assess relevant biomarkers (e.g., certain cytokines) and autoantibodies, and evaluate immunologic, enzymatic, and metabolic parameters as possible post-COVID predictors at different time points, and to provide a basis for further pathophysiological exploration. This approach will allow assessment and identification of the timeline with regard to mechanisms and natural history of the post-COVID syndrome. A modern tool, currently under investigation and explored in COVID-19 condition [75], is multi-dimensional immunoprofiling in combination with machine learning. However, it remains to be seen to what extent this method can contribute to improve understanding of post-COVID syndrome, specifically with regard to predictive validity.

Given the global scale of the pandemic, it is evident that healthcare requirements for patients with sequelae of COVID-19 will remain high or even increase despite large vaccination programs [23]. Thus, with respect to the high prevalence of COVID-19 cases worldwide and current evidence on latent autoimmunity in post-COVID syndrome, it can also be assumed that both cases and spectrum of autoimmune disorders may increase in the near future [17,28].

In fact, only a comprehensive insight and understanding of the pathomechanisms involved in the post-COVID condition will enable proper management of this disease entity. However, adequate patient care cannot be based only on future progress in biomedical research, improvement of infrastructures (including implementation of clinical databases), appropriate financing, and adequate treatment of somatic and mental disorders. Sociological and psychological studies are also needed to better understand the impact of pandemic conditions on somatic and mental disorders along with the post-COVID syndrome.

## Figures and Tables

**Table 1:** Terminology and common clinical definitions of long COVID-19 and post-COVID condition or post-COVID syndrome (completed and updated adaption from [1]).

Origin	Term	Definition
**World Health** **Organization** **(WHO)** [4]	Post COVID-19 condition	Symptoms occurring at least three months after probable or confirmed SARS-CoV-2 infection. Symptoms must last for at least two months and cannot be explained by an alternative diagnosis. Symptoms may be new onset following recovery from acute SARS-CoV-2 infection or persist from the initial illness. Symptoms may fluctuate or relapse.
**National Institute for** **Clinical Excellence (NICE)** [5]	Long COVID (includes ongoing *symptomatic* COVID-19 and post-COVID syndrome)	Symptoms of COVID-19 experienced for 12 or more weeks after initial recovery. Persistence of COVID-19 signs and symptoms that continue to develop after acute COVID-19 and can include both ongoing symptomatic COVID-19 and post-COVID syndrome. **Ongoing symptomatic COVID-19** Signs and symptoms which persist from 4 to 12 weeks after acute COVID-19. **Post COVID-19 syndrome** Signs and symptoms that develop during or after an infection consistent with COVID-19, continue for more than 12 weeks and are not explained by an alternate diagnosis.
**Centers for Disease** **Control and Prevention (CDC)** [6,7]	Post COVID-19 condition	The United States (US) Centers for Disease Control and Prevention uses the term ‘post-COVID conditions’ as an umbrella term for the wide range of health consequences that are present ≥4 weeks after infection with SARS-CoV-2. This includes both general complications of prolonged acute illness and new, returning, or ongoing health problems as post-acute sequelae of SARS-CoV-2 infection (PASC).
**Robert Koch** **Institute (RKI)** [8]	Long COVID-19 vs. post-COVID-19 condition or post-COVID syndrome	Long COVID * Longer-term health impairments following a SARS-CoV-2 infection that are present beyond the acute phase of the sickness of 4 weeks. The symptoms either begin during the acute phase of the disease and persist for a longer period of time, appear or reoccur in the course of weeks and months after the infection. **Post-COVID condition or post-COVID syndrome** Symptoms are either still present at least 12 weeks and longer after the acute infection or appear anew after this period and cannot be explained otherwise.
**Government of Canada** [9]	Post COVID-19 condition	Symptoms of COVID-19 experienced for 12 or more weeks after initial recovery.

* According to the RKI, the term ‘long COVID’ should be used to refer to long-term health consequences of a SARS-CoV-2 infection, as this covers the entire period beyond the acute phase of the disease. For health complaints that explicitly persist for >12 weeks, the term ‘post-COVID condition’ or ‘post-COVID syndrome’ should be used.

**Table 2:** Prevalence of venous thromboembolism. Data from meta-analyses and systematic reviews.

Author (Year)	Pooled Prevalence		
	VTE (95% CI)	DVT (95% CI)	PE (95% CI)
Di Minno et al., [45] (2021)	31.3 (24.3–39.2)	19.8 (10.5–34.0)	18.9 (14.4–24.3)
Leentjens et al., [46] (2021)	14.1 (11.6–16.9)	n.d.	n.d.
Jiménez et al., [47] (2021)	17.0 (13.4–20.9)	12.1 (8.4–16.4)	7.8 (2.6–15.3)
Tan et al., [48] (2021)	14.7 (12.1–17.6)	11.2 (8.4–14.3)	7.8 (6.2 9.4)

Abbreviations: CI, confidence interval; DVT, deep venous thrombosis; PE, pulmonary embolism; VTE, venous thromboembolism, n.d., not determined.

**Table 3:** Risks of autoimmune disorders in a large cohort of patients upon six-month follow-up after acute SARS-CoV-2 infection in comparison to individuals without COVID-19. The statistical analysis is based on propensity scores matching (1:1) for age, sex, race, adverse socioeconomic status, lifestyle-related variables, and comorbidities of more than 880,000 individuals in each cohort. Data are taken from Chang et al. [61].

Autoimmune Disease	Adjusted Hazard Ratio (aHR)	95% Confidence Interval (CI)
Rheumatoid arthritis	2.98	2.78–3.20
Ankylosing spondylitis	3.21	2.50–4.13
Systemic lupus erythematosus	2.99	2.68–3.34
Dermatopolymyositis	1.96	1.47–2.61
Systemic sclerosis	2.58	2.02–3.28
Sjögren’s syndrome	2.62	2.29–3.00
Mixed connective tissue disease	3.14	2.26–4.36
Behçet’s disease	2.32	1.38–3.89
Polymyalgia rheumatica	2.90	2.36–3.57
Vasculitis	1.96	1.74–2.20
Psoriasis	2.91	2.67–3.17
Inflammatory bowel disease	1.78	1.72–1.84
Celiac disease	2.68	2.51–2.85
Diabetes mellitus type 1	2.68	2.51–2.85
**Mortality**	1.20	1.16–1.24

## Data Availability

Not applicable.
